# The development and application of a new tool to assess the adequacy of the content and timing of antenatal care

**DOI:** 10.1186/1472-6963-11-213

**Published:** 2011-09-06

**Authors:** Katrien Beeckman, Fred Louckx, Godelieve Masuy-Stroobant, Soo Downe, Koen Putman

**Affiliations:** 1 Department of Medical Sociology and Health Sciences, Vrije Universiteit Brussel, Faculty of Medicine and Pharmacy, Laarbeeklaan 103, 1090 Brussels, Belgium; 2 Centre de recherche en démographie et société, Université Catholique de Louvain, Place Montesquieu, 1, bte.17 in 1348 Louvain-la-Neuve, Belgium; 3 Research in Childbirth and Health (ReaCH) unit, University of Central Lancashire, Preston, Lancashire, PR1 2HE, UK; 4 Interuniversity Centre for Health Economics Research, Vrije Universiteit Brussel, Faculty of Medicine and Pharmacy, Laarbeeklaan 103, 1090 Brussels, Belgium

## Abstract

**Background:**

Current measures of antenatal care use are limited to initiation of care and number of visits. This study aimed to describe the development and application of a tool to assess the adequacy of the content and timing of antenatal care.

**Methods:**

The Content and Timing of care in Pregnancy (CTP) tool was developed based on clinical relevance for ongoing antenatal care and recommendations in national and international guidelines. The tool reflects minimal care recommended in every pregnancy, regardless of parity or risk status. CTP measures timing of initiation of care, content of care (number of blood pressure readings, blood tests and ultrasound scans) and whether the interventions were received at an appropriate time. Antenatal care trajectories for 333 pregnant women were then described using a standard tool (the APNCU index), that measures the quantity of care only, and the new CTP tool. Both tools categorise care into 4 categories, from 'Inadequate' (both tools) to 'Adequate plus' (APNCU) or 'Appropriate' (CTP). Participants recorded the timing and content of their antenatal care prospectively using diaries. Analysis included an examination of similarities and differences in categorisation of care episodes between the tools.

**Results:**

According to the CTP tool, the care trajectory of 10,2% of the women was classified as inadequate, 8,4% as intermediate, 36% as sufficient and 45,3% as appropriate. The assessment of quality of care differed significantly between the two tools. Seventeen care trajectories classified as 'Adequate' or 'Adequate plus' by the APNCU were deemed 'Inadequate' by the CTP. This suggests that, despite a high number of visits, these women did not receive the minimal recommended content and timing of care.

**Conclusions:**

The CTP tool provides a more detailed assessment of the adequacy of antenatal care than the current standard index. However, guidelines for the content of antenatal care vary, and the tool does not at the moment grade over-use of interventions as 'Inappropriate'. Further work needs to be done to refine the content items prior to larger scale testing of the impact of the new measure.

## Background

The use of antenatal care is considered important in preventing adverse pregnancy outcomes. An association between late initiation of antenatal care or receiving few antenatal visits (< 5) and preterm birth [1-3] or low birth weight [2,4,5] was found in several studies. Other studies however showed that a reduced number of antenatal visits had no influence on birth outcome, as long as effective and appropriate screening, preventive or treatment interventions were taken [6-8].

The term 'adequacy of care' is not uniformly defined. It can include the number of visits [2,9,10], initiation of care [11-13] or continuity of health care provider [14,15]. Furthermore, indices to measure the adequacy of antenatal care trajectories have been conceptualised in different ways. This leads to variations in definitions of the 'adequacy' criteria. The most currently-used indices are the Adequacy of Prenatal Care Index (APNCU) [16,17] and the Graduated Index of Prenatal care Utilization (GINDEX) [18,19]. In both indices, 'adequate care' is defined by the number of consultations adjusted for month when care began and the expected number of visits, adjusted for gestational age at delivery.

Variations in the definition of adequate antenatal care among the different indices lead to different interpretations of results. Furthermore, there is no consensus about the quantity of care a woman should receive [20], especially because one can receive the same content of care in a smaller number of visits [21]. Different authors [19,21] suggested that more comprehensive indices are needed. In addition to measuring the number of visits, qualitative aspects of antenatal care use should also be incorporated, such as indicators of content. Refinement of these indices are likely to result in improved tools for monitoring the care women receive and in better evaluation of compliance with recommended standards. More refined indices should further describe specific antenatal care patterns and provide a more accurate tool to evaluate current health policies and program interventions [22,23].

This study aimed to provide a first step in the development of a more comprehensive tool in which content and timing of antenatal care are considered. The tool was then compared with the currently used APNCU index.

## Methods

### Tool development

#### Selection of indicators to measure content of antenatal care

In order to decide which elements of content of care should be considered, we determined that they needed to be easily and unambiguously identifiable and measurable, and based on the existing evidence for the clinical components of antenatal care that are currently used in many countries and settings. To assess the latter, we looked at the evidence for the following commonly used measures and interventions in pregnancy: evaluation of weight gain; fundal health measurement; routine urine testing for glucose and proteinurea; blood pressure measurement; ultrasound screening for gestational age and for fetal abnormalities; blood tests for anaemia, and for maternal infections that can be transmitted to the fetus/baby. This was not an exhaustive list. At this stage, it was determined that the tool would track three or four key elements of care to see if this altered the definition of adequate care when compared to the standard APNCU measure based on quantity of visits alone.

Thorsdottir et al. [24] demonstrated that evaluation of weight gain during pregnancy can be a predictor of preterm birth, birth weight, macrosomia, large for gestational age babies and small for gestational age (SGA) babies. Further, routinely measuring fundal height seemed not very effective to detect small and large babies [25]. Also the accuracy of routine urine testing for glucose and protein in screening for diabetes or preeclampsia does not seem to be high in clinical practice [26]. In order to detect diabetes, a routine oral 50-g glucose challenge test was superior [26]. Carefully monitoring blood pressure improved the diagnosis and successful treatment of preeclampsia [27].

Other routine interventions such as ultrasound screening in the first trimester are effective in assessing gestational age accurately, in detecting twin pregnancies and, combined with serum screening markers, in screening for Down's syndrome (nuchal translucency scan) [28-30]. An ultrasound scan in the second trimester (18 to 23 weeks) is an effective method to detect structural anomalies [29-31].

Screening for anaemia in pregnancy appears to be a valid intervention, because low and very high levels of haemoglobin are related to increased risks of poor outcome for mother and baby [32-34]. Blood tests to screen for Hepatitis B [35] and Human Immunodeficiency Virus [36] infections are effective and can lead to the prevention of mother-child transmission during childbirth [37], and the initiation of postnatal treatment or vaccination.

To check if these components had widespread support as effective components of antenatal care, we looked at differences in current guidelines between European countries as well as the recommendations of the World Health Organisation and American guidelines. Many guidelines advise the evaluation of weight gain at every visit [38-41], however guidelines are not congruent because some guidelines only suggest measuring weight at the first visit [42,43]. In most guidelines a urine test for proteinuria [39,40,44] is advised at every visit, while some guidelines do not advise checking for proteinuria during pregnancy [38,43]. Measurement of fundal height is not always included in the guidelines [41], and where advised, the timing of commencement varies [38-40,42-44]. Recommendations on screening for gestational diabetes range from universal screening [38], over-screening in some populations [39,40,44] to no screening at all [42,43]. All of these guidelines advise one blood test at the beginning of pregnancy [38-40,43-46]. A second blood screening is advised in most guidelines [39,40,42-44]. Almost all guidelines advise the measurement of blood pressure at every visit [38-40,42,44]. The recommendations on ultrasound use vary between countries. The World Health Organization and the American College of Obstetrics and Gynecologists (ACOG) do not recommend systematic ultrasound in pregnancy unless it is indicated [39,42]. All other guidelines advise at least one ultrasound between weeks 18 and 22 to check for fetal anomalies. Fetal aneuploidy screening between weeks 10 to 13 of gestation is advised in several guidelines [40,43,44].

The previous steps indicated that, despite being nominally based on current best evidence, guidelines are inconsistent. However, after weighing up the clinical evidence and the European guideline recommendation congruence, we decided to focus on blood pressure (BP), blood screening (BS) and ultrasound screening (US) as valid clinical components for the first iteration of the Content and Timing of care in Pregnancy (CTP) tool. We are aware that future iterations of the tool will require a further scrutiny of this evidence, and that this future work would benefit from consensus methods such as a Delphi study among relevant stakeholders. This study was designed to test the feasibility of using such a tool in principle, before undertaking these refinements in future.

#### Timing of care in the CTP and definition of categories

The CTP tool classifies care into a four category ordinal scale; inadequate, intermediate, sufficient or appropriate. The tool aimed to reflect if women received a minimum care package recommended in every pregnancy, regardless of parity or risk status. Given the international consensus that care should start by the time of the fourteenth week of gestation [20,38,44,46], we decided to incorporate timely initiation of care as an element in the tool. We also added the timing of the three chosen interventions during the course of the pregnancy.

In the CTP, care trajectories are first assessed against the timing of initiation of care. Women who first receive care after fourteen completed weeks of gestation are automatically assigned to the inadequate category. The care for the remaining women is then measured against the number of times they receive each element of care over the whole care trajectory. The number of actions for each of the three interventions (US, BP, BS) over the whole pregnancy is calculated. Women join the 'inadequate' category when at least one intervention occurred less than the minimum recommended number of times and the number of the other interventions does not exceed the respective ranges (for example 2 US, 1 BP and 1 BS). When at least one intervention occurred less than the minimum recommended number of times but another exceeded the respective ranges the women is assigned to the intermediate group, for example she received 8 US, 1 BP and 1 BS.

For all women that meet the minimum recommended number of interventions, meaning at least 6 BP and 2 BS and 2 US throughout pregnancy and therefore belonging to the 'sufficient' group, the timing of the interventions in pregnancy is considered. When the minimum number of actions for each intervention all occurred in the relevant trimesters, a woman is classified to the 'appropriate' category. When the time criterion for all three interventions is not fulfilled these women stay in the 'sufficient' group.

Women in the 'appropriate' category received the minimal care package recommended for each pregnancy (independent of risk status or parity). For example, women reaching this stage who, during the first trimester had at least one US, BP and BS, during the second trimester at least one US and two BP measurements, and, during their third trimester, at least three BP measurements and one BS, would be allocated to the 'Appropriate' category.

**Figure 1:**
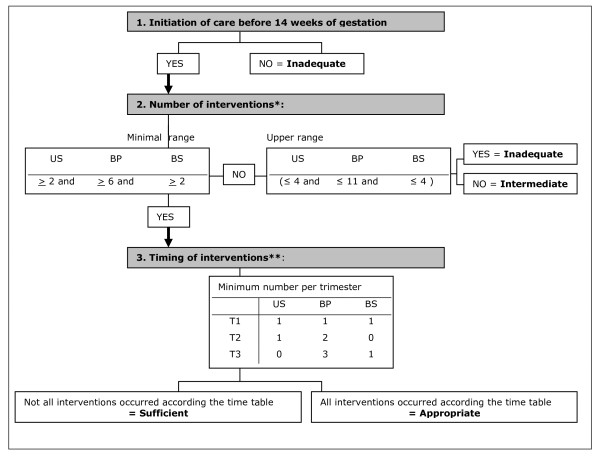
**outline of the Content and Timing of care in Pregnancy (CTP) tool**. US: Ultrasound, BP: Blood Pressure, BS: Blood Screening, T: Trimester. *Ranges: lower value based on the NICE [42] and Belgian guideline [40], upper value based on national data[50]. **based on the NICE[42]. **Inadequate**: initiation of care after first trimester OR the number of at least one intervention is less than the lower range and none of the interventions occurred more than the range. **Intermediate: **initiation of care in the first trimester; the number of at least one intervention occurred less than the lower range and at least one intervention exceeded the range. **Sufficient**: initiation of care in the first trimester; the number of all interventions equals at least the respective lower range but timing of at least one intervention is not as recommended. **Appropriate: **initiation of care in the first trimester; the number of the interventions equals at least the respective lower range and timing of the actions of all basic interventions is as recommended

Figure 1 sets this process out schematically.

### Composition of the APNCU index

The APNCU index was used as the comparator for this study. It takes into account the timing of initiation of care and received number of visits. The index is based on the guidelines for antenatal care use of the ACOG for low risk pregnancies [19]. The APNCU index leads to four categories. The 'inadequate' category includes those women where initiation of care took place after the 4^th ^month (late initiation) or where fewer than 50% of the recommended visits were undertaken. Women starting care before the 4^th ^month and attending between 50-79% of the recommended number of visits are assigned to the 'intermediate' category. Initiation of care before the 4^th ^month and attending 80-109% of the recommended visits assigns women to the 'adequate' group. The 'adequate plus' group contains women starting care before the 4^th ^month of gestation and attending more than 110% of the recommended visits [17]. This measure does not see over-provision as inadequate or inappropriate.

As the current study was undertaken in Belgium, in order to compare the CTP classification with the APNCU index, the APNCU index was adapted from the number of visits recommended by the ACOG to the recommended number of visits within the Belgian guidelines. In this way the effect of the discrepancy between the recommended number of visits in both countries was nullified.

### Setting, study design and participants

In order to map antenatal care use, a prospective observational study was conducted in nine out of eleven medical centres that provide antenatal care in the Brussels Metropolitan Region. In Brussels, irrespective of the type of health care provider that provides antenatal care, all women are referred to one of these centres for their ultrasound scan(s). Women were recruited consecutively between April and July 2008 and included if they were aged over eighteen, residing in the metropolitan region, with a gestational age less of than sixteen weeks, or if they were attending the third antenatal visit or less (visits prior to inclusion were documented at intake). Women were excluded if they had a multiple pregnancy, a medical problem (heart disease, diabetes, hypertension or renal disease for instance), were not reachable by phone or did not give informed consent.

### Data collection

Women were asked to document their ongoing antenatal care by documenting the following for each antenatal visit: place of the visit, person visited, date of the visit, visit scheduled or not, reason for the visit and received interventions during that visit (including weighing, measuring blood pressure, urine test, ultrasound, blood screening, vaginal exam, sugar test,...). A diary was developed to record each antenatal visit in a standardised manner and a protocol was developed to explain to the women how to use the diary. Use of a diary enabled us to record also these interventions next to those made by the regular care provider. Bimonthly telephone follow-up interviews were conducted to record received antenatal care, to reduce recall bias and to verify the completeness of the data. Women had the choice of being called in one of five languages: Dutch, French (two of the official languages), English, Turkish or Arabic (foreign languages currently mostly spoken in Brussels). It was estimated that 95.5% of the population speaks one of those five languages [47]. Intercultural workers conducted the interviews limiting cultural barriers.

We undertook a pilot study (unpublished) that demonstrated that the three interventions selected were easily identified by the women and therefore could be reliably collected via a self-report approach. Data from the pilot study was used as training set to test the algorithm of the CTP tool.

### Data analysis

Characteristics of the study population and their antenatal care use were described using descriptive statistics. Then the number of women in each category for each tool was compared using Chi^2 ^analyses. Data were managed and analyzed with SPSS 17.0.

### Ethical considerations

The principles of the Helsinki Declaration were taken into account. Written, informed

consent was obtained from all participants. The study was approved by all participating sites and by the Ethics Committee of the University Hospital UZ Brussel.

## Results

### Characteristics of the study population and received antenatal care

Complete pregnancy care trajectories were recorded for 333 women. Overall, 79.8% were aged 21-34 years (table 1), 32.1% were of Belgian origin and 30.9% were Maghreb women. Looking at educational level, we found that 14.7% had not finished secondary school. The characteristics of our study sample were compared with the most recent data from the national birth registration available for the Brussels Capital region (N = 16801 in 2007). The data showed no difference for age, educational level, occupational status or origin. Our sample had fewer single mothers (9.3%) compared with the national data (17%) (results available upon request)

**Table 1:** Characteristics of the study population and received antenatal care (N = 333)

Population characteristics		N	%
Age	18-20	14	4.2
	21-35	265	79.8
	> 35	53	16.0
Origin	Belgium	107	32.1
	Maghreb*	103	30.9
	Other	123	36.9
Marital Status	Co-habiting/married	302	90.7
	Single	31	9.3
Educational level	No higher education	199	59.7
	Higher education	134	40.3
Occupational status	Active in the labour market	151	45.3
	Not active in the labour marked	182	54.7
Parity	primiparae	128	38.4
	multiparae	205	61.5

Characteristics of received antenatal care		(mean ± SD)	Median (P25-P75)

Weeks of gestation at initiation of care		8.2 (4.2)	7(6-10)
Total number of antenatal consultations		12.1 (0.2)	11(10-14)
Total number of ultrasounds		5.9 (2.9)	5 (4-7)
Total number of blood samples taken		4.6 (2.2)	8 (6-10)
Total number of blood pressure measurements		7.7(2.8)	8 (6-10)
Weeks of gestation at delivery		39.1 (0.1)	40 (38-40)

SD Standard Deviation

P Percentile

* Maghreb countries: Algeria, Egypt, Libya, Morocco, Mauretania, Tunisia, or Sahara

Table 1 also shows characteristics related to care during pregnancy. Half of the women initiated care at seven weeks of gestation (P25-P75: 6-10), initiation ranged from 0 to 28 weeks of gestation (results not shown). For interventions during pregnancy, we found that half of the women received five ultrasounds (P25-P75: 4-7), eight blood screenings (P25-P75: 6-10) and eight blood pressure measurements (P25-P75: 6-9). Furthermore, half of the women gave birth at 40 weeks of gestation (P25-P75: 38-40). Women in our sample had more ultrasounds (5.9 compared to 4.1) and slightly more blood tests (4.6 compared to 4.1) compared with the latest regional data (2005) [48]. However some interventions, especially the ultrasounds, are not always charged by the health care provider and therefore not included in the national data.

### Comparison of the category distributions for the CTP tool and the APNCU index

**Figure 2:**
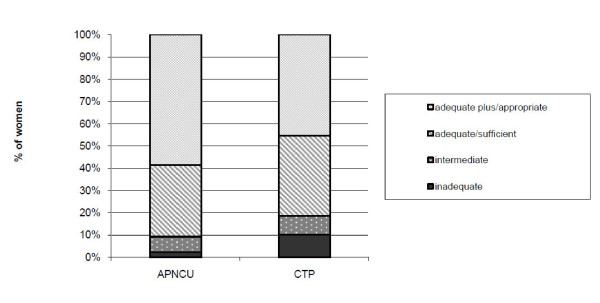
**Distribution of the study sample (N = 333) when using different tools for assessing antenatal care use**. APNCU: Adequacy of Prenatal Care Index. CTP: Content and Timing of care in Pregnancy tool

When considering the antenatal care trajectory, we found that CTP assigned 10.2% of the women into the CTP inadequate care category and 8.4% were assigned as intermediate. Further, 36% and 45.3% of the women were assigned to the CTP sufficient and CTP appropriate care categories respectively (Figure 2). When applying the APNCU index, 2.4% of the women were classified inadequate, 9.6% were assigned to 'intermediate'; while 32.1% of the women were classified 'adequate' and 58.6% were assigned to 'adequate plus'. A significant difference was found when comparing both measures (p < 0.001 (Chi^2 ^test), results not shown).

Examination of the data showed that all eight women in the APNCU inadequate category were also categorised into the CTP inadequate category (Table 2); 80.4% of the APNCU adequate category (86 of 107 women) and 90.3% of the women in the APNCU adequate plus category (176 of 195 women) scored sufficient or appropriate in the CTP classification respectively. However, 21 of 107 women in the APNCU adequate category and 19 of 195 women in the APNCU adequate plus category were assigned to the CTP inadequate or intermediate category. Remarkably, nine women of the APNCU intermediate category were assigned to CTP sufficient or CTP appropriate and another nine cases to CTP inadequate. This allocation can be explained through the number of antenatal visits. The women in the CTP sufficient or CTP appropriate category received between seven and ten visits ie between 70% and 79% of the expected number, while those in the CTP inadequate category received between five and eight visits ie between 50% and 70% of the expected number. APNCU classifies women with 50% to 79% of the expected number of visits and an initiation of care before the 4^th ^month of pregnancy as intermediate, regardless of the content of these visits. Therefore APNCU will give an overestimation of the adequacy of care, while CTP will more closely resemble the actual adequacy of care.

**Table 2:** Cases assigned by CTP tool compared to APNCU

		CTP Classification			
		
APNCU		Inadequate N = 34	Intermediate N = 28	Sufficient N = 120	Appropriate N = 151
Inadequate	(N = 8)	8	0	0	0
Intermediate	(N = 23)	9	5	3	6
Adequate	(N = 107)	9	12	51	35
Adequate Plus	(N = 195)	8	11	66	110

CTP: Content and Timing of care in Pregnancy tool

APNCU: Adequacy of Prenatal Care Index

## Discussion

In this study we developed a tool for assessing antenatal care use that does not only count the number of visits, but also takes into account elements of content and timing of care. As this version of the CTP tool is based on a elements of a basic care package that are recommended by most European guidelines (independent of parity or risk level), the appropriate care category represents women who received the recommended minimum care. The creation of more unambiguous categories is an advantage compared with the APNCU index.

The comparison between the APNCU index and CTP tool showed that, despite the high total number of consultations, women assigned to the APNCU categories 'adequate' or 'adequate plus', were not always classified in the CTP appropriate category. This is because they did not meet the criteria for the number of all three basic interventions combined, or because of inappropriate timing of one of the interventions. The introduction of the additional criteria in the conceptualisation of the CTP tool appeared to lead to a more meaningful approach than with APNCU when assessing the adequacy of antenatal care.

In their review, Alexander and Kotelchuck [19] concluded that it is crucial to understand the conceptual limitations of each quality assessment index in order to make a valid interpretation of the patterns of antenatal care utilisation. The term 'adequacy' has a different meaning in both tools. In the APNCU index, 'adequacy' refers to initiation of care and/or the number of visits controlling for gestational age. In the CTP tool, 'adequacy' not only refers to initiation of care but also to receiving a minimal package of interventions and their timely application throughout the pregnancy (Table 3). With these additional criteria, a more accurate allocation of the care trajectory was possible and therefore the CTP tool may be more relevant in health services research on antenatal care use. The EURO- Peristat group concluded that defining indicators to measure 'content of care' needs further development [20,48]. In this context, CTP might be a first attempt to examine plausible indicators and gain insight in antenatal care differences between countries.

**Table 3:** Comparison of the APNCU and CTP tool

	Tool	
	
	APNCU	CTP
Based on	ACOG	NICE* and National guideline**

Adequate Initiation of Care	Yes	Yes

Adequate number of visits at term gestation	Yes	No

Adequate content of care		

Number and timing of ultrasounds	No	Yes
Number and timing of blood pressure	No	Yes
Number and timing of blood tests	No	Yes

Applicable in high risk pregnancies	No	Yes

APNCU = Adequacy of Prenatal Care Use Index

CTP = Content and Timing of care in Pregnancy tool

ACOG = American College of Obstetricians and Gynecologists [39]

*National institute for Health and Clinical Excellence [44]

**National guideline for prenatal care, Federaal Kenniscentrum voor Gezondheidszorg [40] (Belgian Health Care Knowledge Centre)

Penrod et al. [49] argued for the importance of fully examining the determinants of inadequate antenatal care. In their systematic review, Rowe et al. [50] described the need for further research on the relationship between social inequalities and antenatal care pathways [50]. As the CTP tool includes important items in antenatal pathways, it may be of interest to analyze them across social groups. The usefulness of measuring received care through CTP on birth outcome needs further exploration.

The limitations of the study include the fact that, at this point, the CTP tool focuses on three basic interventions during pregnancy. We are aware that antenatal care encompasses more than these three interventions. Other components of antenatal care such as other clinical dimensions, satisfaction, referral, reason for the intervention, quality of actions undertaken, spacing of visits or behaviour counselling are not included in our tool. Although the tool does account for the adverse effect of overprovision of some aspects of care when there is underprovision of other aspects (in the second iteration of the process) it does not yet account for overuse where other aspects are provided at the minimum level. This aspect would need to be calibrated against clinical need for higher risk women. In our study for example, half of the women received at least five ultrasounds, while recommendations for evidence based practice only advise two ultrasounds [40]. A risk assessment score could be introduced to further fine-tune the CTP tool. We invite other researchers to test the reproducibility and usefulness of the CTP classification in other settings and to further explore indicators reflecting content and quality of antenatal care that can be added to the CTP model. A large prospective research study across Europe, including a Delphi study to refine the essential elements of the tool, might establish a comprehensive standard of measuring adequate and effective antenatal care.

As this was a prospective study, no data on women without antenatal care were available. Although the number of women without antenatal care is low in Western countries [1,51,52] (1% for Belgium [53]), this group is at higher risk for adverse outcome [52]. Because special attention is needed for this particular group we advise creation of a CTP no-care group separate from the inadequate care group.

The use of indices is largely dependent on the data available. For example, Kotelchuck [17] warned about incompleteness of birth certificate data for antenatal care information. The level of detail needed to apply CTP may not be available through standard birth registration forms, requiring additional data collection. However, in some countries, detailed information on antenatal care use is registered, including changes in health care provider (eg the combination of the Medical Birth Register and the data on primary health care visits in Finland, Micronatal^® ^in the Netherlands or the personal medical record in the UK). On the other hand the demand for quality control measures in health care will be accompanied with the recording of different elements of care received. Tools such as the CTP may help decision makers in their choice of what data should be collected in the future.

## Conclusions

Apart from taking into account initiation and elements of content and timing of care, the CTP tool appeared to have some other advantages compared with other indices. Its conceptual framework departs from a basic timing and quantity measure of care recommended in every pregnancy. It reflects the number and timing of three important interventions during pregnancy, resulting in a more detailed picture of antenatal care use. The CTP tool provides a refined judgment on the adequacy of received antenatal care, as aspects of content of care are considered. Therefore, CTP may be useful in studies on determinants of inadequate antenatal care use. CTP needs to be seen as a first step and future work it is needed to develop an even more useful tool, incorporating more of the elements of antenatal care that make a different to the health and wellbeing of childbearing women and their offspring.

### Details Of Ethics Approval

The procedures of the study received ethical approval from the institutional ethics committee responsible for human experimentation. Approval from the ethical committee Academisch Ziekenhuis - Vrije Universiteit Brussel (UZ-Brussel) (University Hospital - VUB) was received on June 29 2006 (2006/084).

## List Of Abbreviations

ACOG: American College of Obstetrics and Gynecologists; APNCU: Adequacy of Prenatal Care Index; BP: Blood pressure; BS: Blood screening; CTP: Content and Timing of care during Pregnancy tool; GINDEX: Graduated Index of Prenatal care Utilization; NICE: National Institute for Health and Clinical Excellence; SGA: small for gestational age; US: Ultrasound scan

## Competing interests

The authors declare that they have no competing interests.

## Authors' contributions

KB contributed to the conception and design of the study, gathered the data, contributed to the analysis and interpretation of data and wrote the article. FL contributed to the conception and design of the study, interpreted the data and revised the article critically for intellectual content. MS contributed to the conception of the study, interpreted the data and revised the article critically for intellectual content. SD contributed to the conception and design of the study, interpreted the data and revised the article critically for intellectual content. KP contributed to the conception and design of the study, participated in the analysis and interpreting of data, and participated in the drafting and revising of the article. All authors read and approved the final manuscript.

## Pre-publication history

The pre-publication history for this paper can be accessed here:

http://www.biomedcentral.com/1472-6963/11/213/prepub

## References

[B1] Blondel B Marshall B Poor antenatal care in 20 French districts: risk factors and pregnancy outcome J Epidemiol Community Health 1998 52 501 506 10.1136/jech.52.8.501 9876361 PMC1756747

[B2] Raatikainen K Heiskanen N Heinonen S Under-attending free antenatal care is associated with adverse pregnancy outcomes BMC Public Health 2007 7 268 10.1186/1471-2458-7-268 17900359 PMC2048953

[B3] Barros H Tavares M Rodrigues T Role of prenatal care in preterm birth and low birthweight in Portugal J Public Health Med 1996 18 321 328 8887844 10.1093/oxfordjournals.pubmed.a024513

[B4] Koroukian SM Rimm AA The "Adequacy of Prenatal Care Utilization" (APNCU) index to study low birth weight: is the index biased? J Clin Epidemiol 2002 55 296 305 10.1016/S0895-4356(01)00471-1 11864801

[B5] Petrou S Kupek E Vause S Maresh M Antenatal visits and adverse perinatal outcomes: results from a British population-based study Eur J Obstet Gynecol Reprod Biol 2003 106 40 49 10.1016/S0301-2115(02)00215-4 12475580

[B6] Villar J Ba'aqeel H Piaggio G Lumbiganon P Miguel BJ Farnot U Al-Mazrou Y Carroli G Pinol A Donner A Langer A Nigenda G Mugford M Fox-Rushby J Hutton G Bergsjø P Bakketeig L Berendes H Garcia J WHO antenatal care randomised trial for the evaluation of a new model of routine antenatal care Lancet 2001 357 1551 1564 10.1016/S0140-6736(00)04722-X 11377642

[B7] Carroli G Villar J Piaggio G Khan-Neelofur D Gulmezoglu M Mugford M Lumbiganon P Farnot U Bersgjø P WHO systematic review of randomised controlled trials of routine antenatal care Lancet 2001 357 1565 1570 10.1016/S0140-6736(00)04723-1 11377643

[B8] Partridge CA Holman JR Effects of a reduced-visit prenatal care clinical practice guideline J Am Board Fam Pract 2005 18 555 560 10.3122/jabfm.18.6.555 16322418

[B9] Walker DS McCully L Vest V Evidence-based prenatal care visits: when less is more J Midwifery Womens Health 2001 46 146 151 10.1016/S1526-9523(01)00120-9 11480746

[B10] Petrou S Kupek E Vause S Maresh M Clinical, provider and sociodemographic determinants of the number of antenatal visits in England and Wales Soc Sci Med 2001 52 1123 1134 10.1016/S0277-9536(00)00212-4 11266054

[B11] Reichman NE Kenney GM Prenatal care, birth outcomes and newborn hospitalization costs: patterns among Hispanics in New Jersey Fam Plann Perspect 1998 30 182 7, 200 10.2307/2991681 9711457

[B12] Hueston WJ Gilbert GE Davis L Sturgill V Delayed prenatal care and the risk of low birth weight delivery J Community Health 2003 28 199 208 10.1023/A:1022908307844 12713070

[B13] Nothnagle M Marchi K Egerter S Braveman P Risk factors for late or no prenatal care following Medicaid expansions in California Matern Child Health J 2000 4 251 259 10.1023/A:1026647722295 11272345

[B14] Boss DJ Timbrook RE Clinical obstetric outcomes related to continuity in prenatal care J Am Board Fam Pract 2001 14 418 423 11757883

[B15] Hodnett ED Continuity of caregivers for care during pregnancy and childbirth Cochrane Database Syst Rev 2000 CD000062 10.1002/14651858.CD000062 10796108

[B16] Kessner DM Singer J Kalk CE Schlesinger ER Infant Death: An Analysis by Maternal Risk and Health Care 1973 Washington DC: Institute of Medicine and National Academy of Sciences Edited by Institute of Medicine and National Academy of Sciences

[B17] Kotelchuck M An evaluation of the Kessner Adequacy of Prenatal Care Index and a proposed Adequacy of Prenatal Care Utilization Index Am J Public Health 1994 84 1414 1420 10.2105/AJPH.84.9.1414 8092364 PMC1615177

[B18] Alexander GR Cornely DA Prenatal care utilization: its measurement and relationship to pregnancy outcome Am J Prev Med 1987 3 243 253 3452362

[B19] Alexander GR Kotelchuck M Quantifying the adequacy of prenatal care: a comparison of indices Public Health Rep 1996 111 408 418 8837629 PMC1381783

[B20] Wildman K Blondel B Nijhuis J Defoort P Bakoula C European indicators of health care during pregnancy, delivery and the postpartum period Eur J Obstet Gynecol Reprod Biol 2003 111 Suppl 1 S53 S65 14642320 10.1016/j.ejogrb.2003.09.006

[B21] Bloch JR Dawley K Suplee PD Application of the Kessner and Kotelchuck prenatal care adequacy indices in a preterm birth population Public Health Nurs 2009 26 449 459 10.1111/j.1525-1446.2009.00803.x 19706128

[B22] Kogan MD Martin JA Alexander GR Kotelchuck M Ventura SJ Frigoletto FD The changing pattern of prenatal care utilization in the United States, 1981-1995, using different prenatal care indices JAMA 1998 279 1623 1628 10.1001/jama.279.20.1623 9613911

[B23] Perloff JD Jaffee KD Prenatal care utilization in New York City: comparison of measures and assessment of their significance for urban health Bull N Y Acad Med 1997 74 51 64 9211001 PMC2359255

[B24] Thorsdottir I Torfadottir JE Birgisdottir BE Geirsson RT Weight gain in women of normal weight before pregnancy: complications in pregnancy or delivery and birth outcome Obstet Gynecol 2002 99 799 806 10.1016/S0029-7844(02)01946-4 11978290

[B25] Neilson JP Symphysis-fundal height measurement in pregnancy Cochrane Database Syst Rev 2000 CD000944 10.1002/14651858.CD000944 PMC7032650 10796225

[B26] Alto WA No need for glycosuria/proteinuria screen in pregnant women J Fam Pract 2005 54 978 983 16266604

[B27] Cnossen JS Vollebregt KC de VN ter RG Mol BW Franx A Khan KS van der Post JA Accuracy of mean arterial pressure and blood pressure measurements in predicting pre-eclampsia: systematic review and meta-analysis BMJ 2008 336 1117 1120 10.1136/bmj.39540.522049.BE 18480117 PMC2386627

[B28] Neilson JP Ultrasound for fetal assessment in early pregnancy Cochrane Database Syst Rev 2000 CD000182 10.1002/14651858.CD000182 10796174

[B29] Whitworth M Bricker L Neilson JP Dowswell T Ultrasound for fetal assessment in early pregnancy Cochrane Database Syst Rev 2010 CD007058 10.1002/14651858.CD007058.pub2 PMC4084925 20393955

[B30] Nicolaides KH Nuchal translucency and other first-trimester sonographic markers of chromosomal abnormalities Am J Obstet Gynecol 2004 191 45 67 10.1016/j.ajog.2004.03.090 15295343

[B31] Saltvedt S Almstrom H Kublickas M Valentin L Grunewald C Detection of malformations in chromosomally normal fetuses by routine ultrasound at 12 or 18 weeks of gestation-a randomised controlled trial in 39,572 pregnancies BJOG 2006 113 664 674 10.1111/j.1471-0528.2006.00953.x 16709209

[B32] Steer PJ Maternal hemoglobin concentration and birth weight Am J Clin Nutr 2000 71 1285S 1287S 10799403 10.1093/ajcn/71.5.1285s

[B33] Zhou LM Yang WW Hua JZ Deng CQ Tao X Stoltzfus RJ Relation of hemoglobin measured at different times in pregnancy to preterm birth and low birth weight in Shanghai, China Am J Epidemiol 1998 148 998 1006 9829872 10.1093/oxfordjournals.aje.a009577

[B34] Rasmussen K Is There a Causal Relationship between Iron Deficiency or Iron-Deficiency Anemia and Weight at Birth, Length of Gestation and Perinatal Mortality? J Nutr 2001 131 590S 601S 11160592 10.1093/jn/131.2.590S

[B35] Lee C Gong Y Brok J Boxall EH Gluud C Effect of hepatitis B immunisation in newborn infants of mothers positive for hepatitis B surface antigen: systematic review and meta-analysis BMJ 2006 332 328 336 10.1136/bmj.38719.435833.7C 16443611 PMC1363909

[B36] Leddy MA Gonik B Schulkin J Obstetrician-gynecologists and perinatal infections: a review of studies of the Collaborative Ambulatory Research Network (2005-2009) Infect Dis Obstet Gynecol 2010 2010 583950 21113289 10.1155/2010/583950 PMC2989373

[B37] Brocklehurst P Volmink J Antiretrovirals for reducing the risk of mother-to-child transmission of HIV infection Cochrane Database Syst Rev 2002 CD003510 10.1002/14651858.CD003510 11869666

[B38] Institute of Clinical Systems Improvement Prenatal Care, Routine (Guideline) 2008 ICSI Institute of Clinical Systems Improvement 26-11-2008. Ref Type: Electronic Citation

[B39] American Academy of Pediatrics the American College of Obstitricians and Gynecologists lockwood CJ Antepartum Care Guidelines for Perinatal Care 2007 Lemons J.A. Washington 83 137

[B40] Lodewyckx K Peeters G Spitz B Blot S Temmerman M Zhang W Alexander S Mambourg F Ramaekers D Nationale richtlijn prenatale zorg: een basis voor een klinisch pad voor de opvolging van zwangerschappen. KCE reports Vol. 6A. Federaal Kenniscentrum voor de Gezondheidszorg 24-12-2004. 26-11-2008. Ref Type: Electronic Citation

[B41] Bernloehr A Smith P Vydelingum V Antenatal care in the European Union: a survey on guidelines in all 25 member states of the Community Eur J Obstet Gynecol Reprod Biol 2005 122 22 32 10.1016/j.ejogrb.2005.04.004 16154036

[B42] WHO Antenatal Care Randomized Trial: Manual for the Implementation of the New Model 2001 World Health Organization 26-11-2008. Ref Type: Electronic Citation

[B43] Richtlijn Basis prenatale zorg. Nederlandse Vereniging voorObstetrie en Gynaecologie 2002 26-11-2008. Ref Type: Electronic Citation

[B44] Antenatal care Routine care for the healthy pregnant woman 2008 National Institute for Health and Clinical Excellence (NICE) 26-11-2008. Ref Type: Electronic Citation

[B45] Family-Centred Maternity and Newborn Care National Guidelines. Care During Pregnancy. Public Health Agency of Canada 2000 26-11-2008. Ref Type: Electronic Citation

[B46] Provision of effective antenatal care 2006 World Health Organization 26-11-2008. Ref Type: Electronic Citation

[B47] Janssens R Van Brussel gesproken Taalgebruik, taalverschuivingen en taalindentiteit in het Brussels Hoofdstedelijk Gewest (Taalbarometer II), Brusselse Thema's 15 2007 Brussel: VUBPRESS

[B48] Zeitlin J, Mohangoo A EURO-PERISTAT project, with SCPE E&E. European Perinatal Health Report. Better statistics for better health for pregnant women and their babies EURO PERISTAT 2008 1 274 Ref Type: Internet Communication

[B49] Penrod JR Lantz PM Measurement error in prenatal care utilization: evidence of attenuation bias in the estimation of impact on birth weight Matern Child Health J 2000 4 39 52 10.1023/A:1009530902429 10941759

[B50] Rowe RE Garcia J Social class, ethnicity and attendance for antenatal care in the United Kingdom: a systematic review J Public Health Med 2003 25 113 119 10.1093/pubmed/fdg025 12848399

[B51] Delvaux T Buekens P Disparity in prenatal care in Europe. Study group on barriers and incentives to prenatal care in Europe Eur J Obstet Gynecol Reprod Biol 1999 83 185 190 10.1016/S0301-2115(98)00237-1 10391530

[B52] Taylor CR Alexander GR Hepworth JT Clustering of U.S. women receiving no prenatal care: differences in pregnancy outcomes and implications for targeting interventions Matern Child Health J 2005 9 125 133 10.1007/s10995-005-4869-3 15965618

[B53] De Gauquier K Remacle A Prenatale zorg in België in 2005. Studie van het Intermutualistisch Agentschap 2007 Brussel, IMA Ref Type: Report

